# Demographic and clinical profile of black patients with chronic kidney disease attending a tertiary hospital in Johannesburg, South Africa

**DOI:** 10.1371/journal.pone.0266155

**Published:** 2022-09-19

**Authors:** Alfred Meremo, Graham Paget, Raquel Duarte, Caroline Dickens, Therese Dix-Peek, Deogratius Bintabara, Saraladevi Naicker

**Affiliations:** 1 Department of Internal Medicine, School of Clinical Medicine, Faculty of Health Science, University of the Witwatersrand, Johannesburg, South Africa; 2 Department of Internal Medicine, School of Medicine & Dentistry, The University Dodoma, Dodoma, Tanzania; 3 Department of Community Medicine, School of Medicine & Dentistry, The University Dodoma, Dodoma, Tanzania; Indiana University Purdue University at Indianapolis, UNITED STATES

## Abstract

**Background:**

The prevalence of chronic kidney disease (CKD) is increasing worldwide; black patients have an increased risk of developing CKD and end stage kidney disease (ESKD) at significantly higher rates than other races.

**Methods:**

A cross sectional study was carried out on black patients with CKD attending the kidney outpatient clinic at Charlotte Maxeke Johannesburg Academic Hospital (CMJAH) in South Africa, *between September 2019 to March 2020*. Demographic and clinical data were extracted from the ongoing kidney outpatient clinic records and interviews, and were filled in a questionnaire. Patients provided blood and urine for laboratory investigations as standard of care, and data were descriptively and inferentially entered into REDcap and analysed using STATA version 17. Multivariable logistic regression analysis was used to identify demographic and clinical variables associated with advanced CKD.

**Results:**

A total of 312 black patients with CKD were enrolled in the study with a median age of 58 (IQR 46–67) years; 58% patients had advanced CKD, 31.5% of whom had grossly increased proteinuria, 96.7% had hypertension, 38.7% had diabetes mellitus and 38.1% had both hypertension and diabetes mellitus. In patients with advanced CKD, the median age was 61 (IQR 51–69) years, eGFR 33 (30–39) mL/min/1.73 m^2^, serum bicarbonate 22 (IQR 20–24), haemoglobin 12.9 (IQR 11.5–14.0) g/dl and serum uric acid 0.43 (IQR 0.37–0.53). The prevalence of metabolic acidosis was 62.4%, anemia 46.4% and gout 30.9% among those with advanced CKD, while the prevalence of metabolic acidosis and anaemia was 46.6% and 25.9% respectively in those with early CKD. Variables with higher odds for advanced CKD after multivariable logistic regression analysis were hypertension (OR 3.3, 95% CI 1.2–9.2, P = 0.020), diabetes mellitus (OR 1.8, 95% CI 1.1–3.3, P = 0.024), severe proteinuria (OR 3.5, 95% CI 1.9–6.5, P = 0.001), angina (OR 2.5, 95% CI 1.2–5.1, P = 0.008), anaemia (OR 2.9, 95% CI 1.7–4.9, P = 0.001), hyperuricemia (OR 2.4, 95% CI 1.4–4.1, P = 0.001), and metabolic acidosis (OR 2.0, 95% CI 1.2–3.1, P = 0.005). Other associations with advanced CKD were loss of spouse (widow/widower) (OR 3.2, 95% CI 1.4–7.4, P = 0.006), low transferrin (OR 2.4, 95% CI 1.1–5.1, P = 0.028), hyperkalemia (OR 5.4, 95% CI 1.2–24.1, P = 0.029), use of allopurinol (OR 2.4, 95% CI 1.4–4.3, P = 0.005) and doxazosin (OR 1.9, 95% CI 1.2–3.1, P = 0.006).

**Conclusion:**

Hypertension and diabetes mellitus were strongly associated with advanced CKD, suggesting a need for primary and secondary population-based prevention measures. Metabolic acidosis, anemia with low transferrin levels, hyperuricemia and hyperkalemia were highly prevalent in our patients, including those with early CKD, and they were strongly associated with advanced CKD, requiring clinicians and dietitians to be proactive in supporting the needs of CKD patients in meeting their daily dietary requirements towards preventing and slowing the progression of CKD.

## Introduction

Chronic kidney disease (CKD), defined as decreased kidney function identified by glomerular filtration rate (GFR) of less than 60 mL/min per 1·73 m^2^, or markers of kidney damage, or both, of at least 3 months duration, regardless of the underlying cause [1], is a major public health issue worldwide and contributes immensely to the overall non-communicable disease (NCD) burden, with NCDs also contributing to the burden of CKD [2, 3]. Chronic kidney disease is usually asymptomatic until the more advanced stages and accurate prevalence data are lacking in most regions including sub-Saharan Africa [4]. As the prevalence of CKD is increasing worldwide and consequently the demand for kidney replacement therapy (KRT), the incidence of cardiovascular events and death is also increasing [5, 6]. Recent systematic reviews have reported the prevalence of CKD to be 15.8% in Africa, similar to other continents, constituting a true public health need with major cost implications to healthcare systems [1, 7]. Diabetes mellitus and hypertension are the leading causes of CKD worldwide; and in sub-Saharan Africa, hypertension is the leading cause of CKD [8, 9]. Studies have shown that African-Americans have a 2- to 4-fold greater risk for end stage kidney disease (ESKD) requiring renal replacement therapy than their white counterparts [10, 11]. Individuals of black ethnicity due to their genetics, including the presence of APOL1 high-risk genotypes, are at higher risk of death due to CKD as a result of social, economic and medical causes [12, 13]. African ancestry has been associated with higher serum creatinine levels, lower eGFR estimates and more rapid CKD progression [14, 15]. In addition to the known risk factors for advanced CKD, which are age, male sex, black race, arterial hypertension and proteinuria, other modifiable factors including medications (traditional and herbal), hyperuricemia, hyperlipidemia, elevated phosphate levels, heart failure and anemia are common in CKD at later stages [16, 17]. Metabolic acidosis increases with worsening eGFR, with prevalence of around 40% among patients with CKD stage 4 and it is associated with rapid CKD progression [18, 19]. Anaemia is common in CKD and is frequently associated with poor outcomes, including increased cardiovascular risks, hospitalization, decreased quality of life and increased risk of mortality [20, 21]. Hyperuricemia greatly contributes to the development of CKD and its progression, it appears that increasing uric acid levels increase the risk for CKD development by causing inflammation, endothelial cell injury and activation of the renin-angiotensin system [22, 23]. Hyperkalemia is also common in advanced CKD; its prevalence increases with decreasing eGFR and it is significantly associated with faster CKD progression [24, 25]. Thus, the aim of this study was to determine the demographic and clinical profile of black patients with CKD attending Charlotte Maxeke Johannesburg Academic Hospital (CMJAH) in Johannesburg, South Africa.

## Methods

### Study design, population and settings

This was a cross-sectional study to evaluate the demographic and clinical profile of black patients with CKD attending the kidney outpatient department (KOPD) clinic at CMJAH between September 2019 to March 2020. The CMJAH is a public accredited central hospital with 1088 beds serving patients from across the Gauteng province and nearby provinces in South Africa, CMJAH is also the main teaching hospital for The University of the Witwatersrand, faculty of Health Sciences. Johannesburg is the largest city in South Africa and among the largest 50 urban agglomerations in the world. Johannesburg had an estimated population of around 5.9 million in 2021, the most common racial groups include; black African (76.4%), colored (5.6%), White (12.3%) and Indian/Asian (4.9%).

Inclusion criteria included patients who were >18 years of age, CKD stages 1–4, who had controlled hypertension (blood pressure < 140/90 mm Hg) and diabetes mellitus (HbA1C < 7%), attending the KOPD clinic for at least 6 months and were able to provide informed consent. Patients who had active infections, active malignancies, autoimmune diseases and who were not black were excluded, black patients have an increased risk of developing CKD and end stage kidney disease (ESKD) at significantly higher rates than other races [13, 15].

### Data collection and laboratory procedures

Demographic and clinical data including age, gender, weight, height, glycemic status, history of smoking, etiology of CKD and medications were extracted from the ongoing continuous KOPD clinic records and face to face interviews, and were filled in a questionnaire. Systolic and diastolic blood pressure was measured 3 times, the average of the second and third measurements was used. Body mass index (BMI) was calculated using the National Health Services (NHS-UK) BMI calculator [26]. Measurements of urinary protein creatinine ratio (uPCR), serum creatinine, electrolytes, HbA1C, WBC, haemoglobin level, platelets, calcium, phosphate, transferrin and HDL cholesterol were done as standard of care at the time of recruitment during a clinic visit. Serum creatinine was measured using the isotope dilution mass spectrometry (IDMS) traceable enzymatic assay and estimated glomerular filtration rate (eGFR) was calculated using the Chronic Kidney Disease Epidemiology Collaboration (CKD-EPI) equation without using the African American correction factor [27]. Patients with eGFR < 45 ml/min/1.72m^2^ were considered to have advanced CKD, while those patients with eGFR ≥ 45 ml/min/1.72m^2^ early CKD.

### Data management and analysis

Study data were collected and entered into REDCap (Research Electronic Data Capture) tools [28, 29] hosted at the University of the Witwatersrand and analyzed using STATA version 17 (College Station, Texas, USA). Descriptive statistics were used to summarize demographic and clinical characteristics; continuous variables have been reported as medians with interquartile ranges and Wilcoxon rank-sum test was used for the non-normally distributed variables. Discrete variables have been reported as frequencies and proportions, Pearson’s chi-square test were used to test for association between two variables. Odd ratios were used to estimate the strength of association between variables and advanced CKD, univariate and multivariate logistic regression models have been used to estimate the association of variables and advanced CKD. Variables with p-value less than 0.2 on univariate logistic regression models were then fitted into the multivariate logistic regression models with the addition of age and sex as adjusting variables; variables with a p-value of less than 0.05 were considered to have significant strength of association.

### Ethical issues

Ethical approval was obtained from the Human Research Ethics Committee of the University of Witwatersrand, Johannesburg (ethics clearance certificate No. M190553). Written informed consent was obtained from each of the participants before embarking on data collection.

## Results

### Demographic and clinical characteristics of the study population

Of the 476 black patients with CKD stages 1–4, 164 patients were excluded from the study including 110 patients who had uncontrolled hypertension, 35 patients who had uncontrolled diabetes mellitus, 11 patients had autoimmune diseases, 6 patients had active infections and 2 patients had active malignancies. A total of 312 CKD black patients were enrolled into this study, of whom 162 (51.9%) were male, 292 (93.6%) were hypertensive, 103 (33.0%) were diabetic and 164 (52.6%) were married. The median age was 61 (IQR 51–69) years for advanced CKD and 53 (IQR 41–62) years for early CKD; the median eGFR was 33 (30–39) mL/min/1.73 m^2^ for advanced CKD and 60 (IQR 51–75) mL/min/1.73 m^2^ for early CKD; the median urine protein creatinine ratio (uPCR) was 0.029 (IQR 0.015–0.67) g/mmol for advanced CKD and 0.016 (IQR 0.008–0.034) g/mmol for early CKD. The median serum bicarbonate was 22 (IQR 20–24) mmol/L for advanced CKD and 23 (IQR 21–25) mmol/L for early CKD; the median haemoglobin (Hb) was 12.9 (IQR 11.5–14.0) g/dl for advanced CKD and 13.8 (IQR 12.4–15.7) g/dl for early CKD; the median serum transferrin was 2.44 (IQR 2.23–2.73) g/L for advanced CKD and 2.62 (IQR 2.37–2.89) g/L for early CKD. The median serum uric acid was 0.43 (IQR 0.37–0.53) mmol/L for advanced CKD and 0.36 (IQR 0.30–0.46) mmol/L for early CKD (Table 1).

**Table 1:** Demographic characteristics and clinical profile of 312 CKD patients by eGFR.

Characteristic	eGFR < 45 ml/min/1.72m^2^ (n = 181)	eGFR ≥ 45 ml/min/1.72m^2^ (n = 131)	P-value
	Proportion (%) or Median (IQR)	Proportion (%) or Median (IQR)	
Age (years)	61 (51–69)	53 (41–62)	**0.001**
**Sex**			
Male	96 (53.0%)	66 (50.4%)	
Female	85(47.0%)	65 (49.6%)	0.643
**Marital status**			
Single	43(23.8%)	48(36.6%)	
Married	98 (54.1%)	66 (50.4%)	
Widow/Widower	30(16.6%)	10 (7.6%)	
Separated/Divorced	10(5.5%)	7 (5.4%)	**0.026**
**Highest level of education**			
No formal education	24(13.3%)	14 (10.7%)	
Primary	41(22.7%)	30 (22.9%)	
Secondary	56(30.9%)	43 (32.8%)	
Tertiary	60(33.1%)	44 (33.6%)	0.505
**Occupation**			
Unemployed	26(14.4%)	19(14.5%)	
Domestic workers	37(20.4%)	25(19.1%)	
Self employed	43(23.8%)	27(20.6%)	
Public / Private servant	57(31.5%)	52(39.7%)	
Retired	18(9.9%)	8(6.1%)	0.523
BMI (kg/m^2^)	30.2 (26.0–34.5)	30.2 (26.6–34.7)	0.731
SBP (mmHg)	140 (130–140)	140 (128–140)	0.339
DBP (mmHg)	82 (72–90)	83 (74–90)	0.630
uPCR (g/mmol)	0.029 (0.015–0.67)	0.016 (0.008–0.034)	**0.001**
Creatinine (umol/L)	163 (141–190)	109 (88–122)	**0.001**
eGFR (ml/min/1.72m^2^)	33 (30–39)	60 (51–75)	**0.001**
FBG (mmol/L)	4.5 (4.2–5.2)	4.4 (4.2–4.9)	0.146
HbA1C (%)	7.0 (6.6–7.0)	7.0 (7.0–7.0)	0.222
Haemoglobin (g/dl)	12.9 (11.5–14.0)	13.8 (12.4–15.7)	**0.001**
Transferrin (g/L)	2.44 (2.23–2.73)	2.62 (2.37–2.89)	**0.001**
WBC (x 10^9^ cells/L)	6.4 (5.13–7.71)	6.03 (4.81–7.73)	0.420
Platelets (x 10^9^ cells/L)	256 (213–320)	271 (217–325)	0.378
Uric acid (mmol/L)	0.43 (0.37–0.53)	0.36 (0.30–0.46)	**0.001**
HDL cholesterol (mmol /L)	1.23 (0.98–1.48)	1.22 (1.02–1.52)	0.528
Calcium (mmol /L)	2.31 (2.23–2.40)	2.33 (2.26–2.42)	0.054
Phosphate (mmol /L)	1.1 (0.94–1.24)	1.02 (0.85–1.15)	**0.005**
Sodium (mmol/L)	141 (138–143)	141 (139–143)	0.965
Potassium (mmol/L)	4.4 (3.9–4.8)	4.1 (3.9–4.4)	**0.001**
Bicarbonate (mmol/L)	22 (20–24)	23 (21–25)	**0.013**

IQR, interquartile range; uPCR, urine protein creatinine ratio; DBP, diastolic blood pressure; eGFR, estimated glomerular filtration rate; FBG, fasting blood glucose; HbA1C, glycosylated hemoglobin A1C; WBC, white blood cells; HDL, high density lipoprotein; SBP, systolic blood pressure.

### Clinical profile of CKD patients

Of the 312 CKD black patients, 58% patients had advanced CKD (CKD stage 3b or 4), of whom 57 (31.5%) patients presented with severely increased proteinuria as compared to 23 (17.4%) patients with early CKD. Metabolic acidosis was present in 113 (62.4%) of those who had advanced CKD and in 61 (46.6%) patients with early CKD. Anaemia was present in 84 (46.4%) patients with advanced CKD including 30 (16.6%) patients who had low transferrin levels, while 34 (25.9%) patients with early CKD had anemia. Among patients with advanced CKD, hyperuricemia was found in 56 (30.9%) patients and 16 (8.8%) patients had hyperkalemia. Among patients with advanced CKD, majority (96.7%) patients were diagnosed with hypertension, 70 (38.7%) patients had diabetes mellitus and 69 (38.1%) had both hypertension and diabetes mellitus as compared to 117 (89.3%) patients who had hypertension, 33 (25.2%) patients who had diabetes mellitus and 32 (24.4%) had both hypertension and diabetes mellitus among those with early CKD. Angina was reported in 41 (22.7%) patients with advanced CKD and 15 (11.5%) patients in early CKD. Most (56.9%) patients with advanced CKD were using more than 5 medications as compared to 41.2% patents with early CKD who were using 3–4 medications for their blood pressure control. Majority (84.0%) of the patients with advanced CKD and 76.3% patients in early CKD were on calcium channel blockers (CCBs), while 18.8% patients with advanced CKD and 21.4% of those with early CKD were using angiotensin converting enzyme inhibitors (ACEIs) or angiotensin receptors blockers (ARBs). For patients with advanced CKD, most (57.5%) were on diuretics followed by 51.4% on doxazosin; 29.3% of patients were on insulin and 16 (8.8%) on oral hypoglycemic agents (Table 2).

**Table 2:** Clinical profile of 312 CKD patients by eGFR.

Parameter	eGFR < 45 ml/min/1.72m^2^ (n = 181) Proportion (%)	eGFR ≥ 45 ml/min/1.72m^2^ (n = 131) Proportion (%)	P-value
**eGFR (ml/min/1.72m** ^**2**^ **)**			
Stage 1 (> 90)	0 (0.0%)	4 (3.1%)	
Stage 2 (60–89)	0 (0.0%)	62 (47.3%)	
Stage 3a (45–59)	0 (0.0%)	65 (49.6%)	
Stage 3b (30–44)	144 (79.6%)	0 (0.0%)	
Stage 4 (16–29)	37 (20.4%)	0 (0.0%)	**0.001**
**Diagnoses encountered**			
Hypertension	175 (96.7%)	117 (89.3%)	**0.009**
Diabetes mellitus	70 (38.7%)	33 (25.2%)	**0.012**
Hypertension & Diabetes mellitus	69 (38.1%)	32 (24.4%)	**0.011**
Adult polycystic kidney disease	5 (2.8%)	7 (5.3%)	0.242
Reflux nephropathy	0 (0.0%)	5 (3.8%)	**0.008**
Obstructive uropathy	1 (0.6%)	0 (0.0%)	0.394
Unknown	6 (3. 3%)	5 (3.8%)	0.812
**Current smoking**			
Yes	13 (7.2%)	10 (7.6%)	
No	168 (92.8%)	121 (92.4%)	0.880
**Current Alcohol**			
Yes	14 (7.7%)	18 (13.7%)	
No	169 (92.3%)	113 (86.3%)	0.084
**Cardiovascular Diseases**			
None	120 (66.3%)	108 (82.4%)	
Angina	41 (22.7%)	15 (11.5%)	
Myocardial infarction	8 (4.4%)	4 (3.0%)	
Heart failure	6 (3.3%)	2 (1.5%)	
Stroke	6 (3.3%)	1 (0.8%)	
Transient ischemic attack	0 (0.0%)	1 (0.8%)	**0.017**
**Medications**			
None	2 (1.1%)	8 (6.1%)	**0.004**
Diuretics	104 (57.5%)	58 (44.3%)	**0.021**
ACEIs / ARBs	34 (18.8%)	28 (21.4%)	0.572
Aldactone	6 (3.3%)	5 (3.8%)	0.812
CCBs	152 (84.0%)	100 (76.3%)	0.092
Statins	100 (55.3%)	60 (45.8%)	0.099
Oral hypoglycemics	16 (8.8%)	21(16.0%)	0.053
Insulin	53 (29.3%)	19 (14.5%)	**0.002**
Allopurinol	22 (12.1%)	13 (9.9%)	0.538
Junior ASA	90 (49.7%)	57 (43.5%)	0.278
Beta blockers	41 (22.7%)	30 (22.9%)	0.959
Aldomet	12 (6.6%)	7 (5.3%)	0.639
Hydralazine	4 (2.2%)	3 (2.3%)	0.962
Doxazosin	93 (51.4%)	48 (36.6%)	**0.010**
Others	68 (37.6%)	38 (29.0%)	0.115
**Number of medications per patient**			
0	2 (1.1%)	8 (6.1%)	
1–2	20 (11.1%)	20 (15.3%)	
3–4	56 (30.9%)	55 (41.2%)	
≥ 5	103 (56.9%)	48 (36.6%)	**0.001**
**uPCR (g/mmol)**			
Normal to mildly increased (<0.015)	47 (26.0%)	63 (48.2%)	
Moderately increased (0.015–0.05)	77 (42.5%)	45 (34.4%)	
Severely increased (> 0.050)	57 (31.5%)	23 (17.4%)	**0.001**
**Haemoglobin (g/dl)**			
Normal (> 12.0 or 13.0)	97 (53.6%)	97 (74.1%)	
Anaemia (<12.0 or 13.0)	84 (46.4%)	34 (25.9%)	**0.001**
**Transferrin g/dl)**			
Low (< 2.0)	30 (16.6%)	10 (7.6%)	
Normal (2.0–3.60)	148 (81.8%)	117 (89.3%)	
High (>3.60)	3 (1.7%)	4 (3.1%)	0.052
**Uric acid (mmol/l)**			
Low (< 0.16 or 0.21)	4 (2.2%)	15 (11.5%)	
Normal (0.16/0.21–0.36/0.43)	121 (66.9%)	98 (74.8%)	
High (> 0.36 or 0.43)	56 (30.9%)	18 (13.7%)	**0.001**
**Potassium (mmol/l)**			
Low (< 3.5)	13 (7.2%)	15 (11.5%)	
Normal (3.5–5.1)	152 (84.0%)	114 (87.0%)	
High (> 5.1)	16 (8.8%)	2 (1.5%)	**0.013**
**Bicarbonate (mmol/l)**			
Low (< 23)	113 (62.4%)	61 (46.6%)	
Normal (23–29)	67 (37.0%)	68 (51.9%)	
High (> 29)	1 (0.6%)	2 (1.5%)	**0.018**

uPCR, urine protein creatinine ratio; eGFR, estimated glomerular filtration rate; CCBs, calcium channel blockers; ACEIs, angiotensin converting enzyme inhibitors; ARBs, Aldosterone receptors blockers.

### Factors associated with advanced CKD

We divided the patients into two subgroups according to their CKD stages [early CKD (eGFR ≥ 45 ml/min/1.72m^2^) vs. advanced CKD (eGFR < 45 ml/min/1.72m^2^)]. A total of 24 potential variables were identified after performing univariate logistic regression analyses. Backward elimination reduced this to 15 parameters; the factors associated with advanced CKD after adjusting for age and sex on multivariate logistic regression analysis included: hypertension (OR 3.3, 95% CI 1.2–9.2, P = 0.020), diabetes mellitus (OR 1.8, 95% CI 1.1–3.3, P = 0.024), angina (OR 2.5, 95% CI 1.2–5.1, P = 0.008), severe proteinuria (OR 3.5, 95% CI 1.9–6.5, P = 0.001), moderate proteinuria (OR 2.5, 95% CI 1.5–4.3, P = 0.001), hyperuricemia (OR 2.4, 95% CI 1.4–4.1, P = 0.001), anaemia (OR 2.9, 95% CI 1.7–4.9, P = 0.001), metabolic acidosis(OR 2.0, 95% CI 1.2–3.1, P = 0.005), allopurinol (OR 2.4, 95% CI 1.4–4.3, P = 0.005) and doxazosin (OR 1.9, 95% CI 1.2–3.1, P = 0.006), low transferrin (OR 2.4, 95% CI 1.1–5.1, P = 0.028), hyperkalemia (OR 5.4, 95% CI 1.2–24.1, P = 0.029) and, widow/widower (OR 3.2, 95% CI 1.4–7.4, P = 0.006) (Table 3).

**Table 3:** Factors associated with advanced CKD.

Characteristic	Number of patients	UNIVARIATE		MULTIVARIATE	
		OR (95% CI)	P. value	OR (95% CI)	P. value
**Age**	312	1.1(1.0–1.2)	0.001		
**Marital status**					
Single	91	1(Reference)		1(Reference)	
Married	164	1.7 (0.9–2.8)	0.055	1.5 (0.9–3.0)	0.137
Widow/Widower	40	3.3 (1.5–7.7)	0.004	3.2 (1.4–7.4)	**0.006**
Separated/Divorced	17	1.6 (0.6–4.6)	0.038	1.4 (0.5–4.3)	0.466
**Hypertension**					
No	20	1(Reference)		1(Reference)	
Yes	292	3.5 (1.3–9.3)	0.013	3.3 (1.2–9.2)	**0.020**
**Diabetes mellitus**					
No	209	1(Reference)		1(Reference)	
Yes	103	1.9 (1.1–3.1)	0.013	1.8 (1.1–3.0)	**0.024**
**Current Alcohol**					
No	280	1(Reference)		1(Reference)	
Yes	32	0.5 (0.3–1.1)	0.088	0.5 (0.2–1.0)	0.059
**Cardiovascular Disease:**					
None	228	1(Reference)		1(Reference)	
Angina	56	2.7 (1.4–5.3)	0.004	2.5 (1.2–5.1)	**0.008**
Myocardial infarction	12	1.8 (0.5–6.1)	0.348	1.4 (0.4–5.0)	0.578
Heart failure	8	2.7 (0.5–13.7)	0.230	2.8 (0.5–14.5)	0.219
Stroke	8	5.4 (0.6–45.6)	0.121	4.6 (0.5–39.2)	0.167
**Medications**					
No	10	1 (Reference)		1(Reference)	
Yes	302	11.7 (1.4–94.8)	0.021	0.1 (0.0–0.9)	**0.042**
**Diuretics**					
No	150	1(Reference)		1(Reference)	
Yes	162	1.7 (1.1–2.7)	0.022	1.6 (1.0–2.5)	0.053
**CCB**					
No	60	1(Reference)		1(Reference)	
Yes	252	1.6 (0.9–2.9)	0.093	1.6 (0.9–2.8)	0.127
**Statins**					
No	152	1(Reference)		1(Reference)	
Yes	160	1.5 (0.9–2.3)	0.100	1.4 (0.9–2.2)	0.171
**Oral hypoglycemics**					
No	275	1(Reference)		1(Reference)	
Yes	37	0.5 (0.3–1.0)	0.056	0.5 (0.2–1.0)	**0.048**
**Allopurinol**					
No	277	1(Reference)		1(Reference)	
Yes	35	2.5 (1.4–4.4)	0.003	2.4 (1.3–4.3)	**0.005**
**Nitrates**					
No	306	1(Reference)		1(Reference)	
Yes	6	1.9 (1.1–3.3)	0.024	1.7 (1.0–3.0)	0.059
**Doxazosin**					
No	171	1(Reference)		1(Reference)	
Yes	141	1.8 (1.2–2.9)	0.010	1.9 (1.2–3.1)	**0.006**
**Others**					
No	206	1(Reference)		1(Reference)	
Yes	106	1.5 (0.9–2.4)	0.116	1.5 (0.9–2.5)	0.100
**Proteinuria (g/mmol)**					
Normal to mild (<0.015)	110	1(Reference)		1(Reference)	
Moderately (0.015–0.05)	122	2.3 (1.4–3.9)	0.002	2.5 (1.5–4.3)	**0.001**
Severely (> 0.050)	80	3.9 (2.0–7.2)	0.000	3.5 (1.9–6.5)	**0.001**
**Hyperuricemia**					
No	216	1(Reference)		1(Reference)	
Yes	96	2.4 (1.4–4.0)	0.001	2.4 (1.4–4.1)	**0.001**
**Anaemia**					
No	194	1(Reference)		1(Reference)	
Yes	118	2.5 (1.5–4.0)	0.001	2.9 (1.7–4.9)	**0.001**
**Transferrin g/dl)**					
Normal (2.0–3.60)	265	1(Reference)		1(Reference)	
Low (< 2.0)	40	2.4 (1.1–5.1)	0.025	2.4 (1.1–5.1)	**0.028**
High (>3.60)	7	0.6 (0.1–2.7)	0.499	0.5 (0.1–2.7)	0.491
**WBC (x 10**^**9**^ **cells/l)**					
Normal (4–11)	276	1(Reference)		1(Reference)	
Low (> 4)	23	0.5 (0.2–1.3)	0.051	1.8 (0.7–4.2)	0.202
High (>11)	13	1.1 (0.4–3.5)	0.859	1.8 (0.4–7.5)	0.418
**Calcium (mmol /l)**					
Normal (2.15–2.45)	244	1(Reference)		1(Reference)	
Low (< 2.15)	25	2.2 (0.9–5.8)	0.098	2.1 (0.8–5.8)	0.125
High (>2.45)	43	0.6 (0.3–1.1)	0.081	0.6 (0.3–1.1)	0.081
**Potassium (mmol/l)**					
Normal (3.5–5.1)	266	1(Reference)		1(Reference)	
Low (< 3.5)	28	0.7 (0.3–1.4)	0.280	0.6 (0.3–1.4)	0.261
High (> 5.1)	18	6.0 (1.4–26.6)	0.018	5.4 (1.2–24.1)	**0.029**
**Bicarbonate (mmol/l)**					
Normal (23–29)	135	1(Reference)		1(Reference)	
Low (< 23)	174	1.9 (1.2–3.0)	0.007	2.0 (1.2–3.1)	**0.005**
High (> 29)	3	0.5 (0.1–5.7)	0.583	0.4 (0.0–5.1)	0.495

P < 0.05 was used to identify potential variables, those with P <0.2 in univariate were included into the multivariate analysis adjusting for age and sex.

## Discussion

This study evaluated the demographic and clinical profile of black patients with CKD attending the CMJAH kidney outpatient clinic in Johannesburg, South Africa. There were 42% with early CKD and 58% with advanced CKD; 93.6% had hypertension and 33.0% diabetes mellitus, and 32.3% had both hypertension and diabetes. The prevalence of hypertension among patients with CKD is high and it is strongly associated with advanced CKD and CKD progression [30, 31], the majority (96.7%) of patients with advanced CKD had hypertension, similar to findings in other studies from SSA [9, 32]. Peripherally acting α-blockers like doxazosin are commonly used in the management of hypertension in CKD, mainly due to their pharmacokinetic profile that is undisturbed by worsening kidney function and their role in blood sugar control [33, 34], approximately 51.4% of patients with advanced CKD were using doxazosin for treatment of hypertension with a 1.9 times increased association with advanced CKD, as also has been reported in other studies [35, 36]. Studies have shown that diabetes related CKD is the leading cause of end stage kidney disease among patients with T2DM patients worldwide [37, 38], approximately 38.7% patients with advanced CKD had T2DM; T2DM had 1.8 increased risk for advanced CKD, similar to other studies conducted among black patients in South Africa and Ethiopia [32, 39]. In patients with advanced CKD, approximately 29.3% of the patients were on insulin and 8.8% were on oral hypoglycemics for treatment of their T2DM. Oral hypoglycemics were associated with 0.5 times higher risk for advanced CKD, similar to other studies [40, 41].

Metabolic acidosis is common in CKD and it can lead to dysfunction of many organs and systems including the kidney resulting in CKD progression [42, 43], the prevalence of metabolic acidosis was 62.4% in advanced CKD and 46.6% in early CKD; this prevalence is higher than the 33% - 40% among patients with CKD stage 3–4 from other continents [43–45]. The possible explanation could be firstly, the more rapid CKD progression which has been shown to occur in black patients even early in their CKD stages [15, 19] and secondly, diet where replacement of traditional diets with contemporary/ western foods which contain mainly animal proteins, less vegetables and low intake of fruits might increase CKD patients’ dietary acid load [46, 47]. Patients with low serum bicarbonate levels were 2-fold more likely to have advanced CKD, as reported also in other studies [18, 44]. Anaemia is common in CKD, and it is associated with decreased quality of life, high morbidity and mortality [48, 49]. The prevalence of anaemia was 46.4% in advanced CKD, including 16.6% who had low transferrin levels, while 25.9% of the patients with early CKD had anaemia, advanced CKD was 2.9 times more prevalent if a patient had anemia and 2.4 times more prevalent if a patient had low transferrin, similar to other studies [20, 21, 50]. High serum uric acid levels are associated with high risk for advanced CKD and CKD progression [51, 52], the prevalence of hyperuricemia was 30.9% in advanced CKD; advanced CKD had a 2.4-fold higher OR if a patient had hyperuricemia and 2.4-fold higher if a patient was on allopurinol; similar findings have been reported in other studies [23, 53]. Hyperkalemia is common in patients with chronic CKD and its prevalence increases as the eGFR declines [54, 55], approximately 8.8% patients with advanced CKD were found to have hyperkalemia; hyperkalemia had a 5.4-fold increased association with advanced CKD; similar findings have been reported in other studies [24, 25].

Studies have shown that the incidence of cardiovascular events increases with worsening kidney function [56, 57]; 22.7% patients with advanced CKD reported angina with a 2.5 times increased risk in advanced CKD, similar to other studies [56, 58]. Also 31.5% patients with advanced CKD presented with severely increased proteinuria; advanced CKD was 3.5 times higher if a patient had severe proteinuria, as also reported in other studies [39, 59, 60]. Furthermore, few (18.8%) patients with advanced CKD and 21.4% of those with early CKD were using ACEIs or ARBs; similar findings have been reported from other studies on the underutilization of ACEIs/ARBs when they were clinically indicated or discontinuation of RAAS inhibitors by clinicians during the course of CKD possibly due to their associated side effects [61–63]. Angiotensin converting enzyme inhibitors (ACEIs) or angiotensin receptors blockers (ARBs) had no significant association with advanced CKD, possibly due to the small numbers on these agents, unlike findings from other studies that did demonstrate that the use of ACEIs/ARBs had beneficial effects for kidney events and cardiovascular outcomes compared to other antihypertensive medications in patients with CKD [63–65]. The possible explanation could be the fact that the majority (84.0%) of the study patients with advanced CKD were using calcium channel blockers. Studies have demonstrated that calcium channel blockers have similar kidney and cardiovascular protective effects when compared to RAAS blockers in patients with CKD [66, 67].

## Conclusion

Hypertension and diabetes mellitus were strongly associated with advanced CKD, suggesting a need for primary and secondary population-based prevention measures. Metabolic acidosis, anaemia with low transferrin levels, hyperuricemia and hyperkalemia were highly prevalent in our patients, including those with early CKD, and they were strongly associated with advanced CKD. This calls for the proactive role of clinicians and dietitians in supporting the needs of CKD patients in meeting their daily dietary requirements towards preventing and slowing the progression of CKD. Further studies on the role of diet including plant-based proteins, vegetables and fruits in preventing and slowing CKD progression and other metabolic complications of CKD are warranted.

## Abbreviations

CKD: chronic kidney diseases
CMJAH: Charlotte Maxeke Johannesburg Academic Hospital
CKD-EPI: chronic kidney disease epidemiology collaboration
ESKD: end stage kidney disease
T2DM: Type 2 Diabetes Mellitus
eGFR: estimated glomerular filtration rate
IDMS: isotope dilution mass spectroscopy
uPCR: urine protein creatinine ratio
CCBs: calcium channel blockers
ACEIs: angiotensin converting enzyme inhibitors
ARBs: Aldosterone receptors blockers
KDIGO: kidney disease improving global outcomes
NCDs: non-communicable diseases
SSA: sub-Saharan Africa
